# 

**Published:** 1906-09-22

**Authors:** 


The Worshipful the Mayor of West Ham has kindly consented to preside at the annual dinner of the West Ham and East London Hospital, which is to be held in the Hamilton Hall, Great Eastern Hotel, on October 11th next. Up to the present the following gentlemen have kindly consented to act as stewards: The Lord Claud J. Hamilton; R. A. Gillespie, Esq.; H. E. Lester, Esq., J.P.; F. W. F. Clark, Esq.; F. Fredericks, Esq.; the Lord Kinnaird; Alex. Ritchie, Esq., J.P., C.C.; Thos. Alex. Cook, Esq.; A. Carter, Esq. Situated in the far east of London, in the centre of a poor and densely populated district, the claims of this deserving hospital do not come under the notice of the more favoured parts of the Metropolis, and as a result the Committee experience the greatest difficulty in carrying on the good work of the hospital. The managers of the King's Hospital Fund have reported that the hospital is economically worked and does much good in a very poor district. The hospital is primarily an accident hospital, but the large number of medical cases requiring attention have made an addition to the present buildings an absolute necessity, and funds are urgently needed.

### NOTICE TO CORRESPONDENTS.

**All MSS., Letters, Books for Review, and other matters intended for the Editor should be addressed The Editor, "The Hospital," The Hospital Buildings, 28 & 29 Southampton Street, Strand, W.C.**

**The Editor cannot undertake to return rejected MS., even when accompanied by stamped directed envelope.**

### Notice to Advertisers and Subscribers.

**All Advertisements, Orders for Copies of The Hospital, and Business Communications should be addressed to THE MANAGER (Not to the Editor), The Hospital, 28 & 29 Southampton Street, Strand London, W.C.**

**The Cost of Subscription, post free, is as follows:—Per week, 3½d.; per quarter, 3s. 3d.; per half-year, 6s. 6d.; per annum, 13s. Foreign and Colonial:—Per annum, 19s. 6d., payable, in all cases, in advance.**

### The OCTOBER C.M.B. EXAM.

**Nurses entering for the October Examination of the Central Midwives Board will find the following Books on Midwifery of the greatest assistance in acquiring a thoroughly well-grounded theoretical knowledge of the subject.**

### A COMPLETE HANDBOOK OF MIDWIFERY

By J. K. Watson, M.D. Edin., Author of "A Handbook for Nurses."
350 pp., profusely illustrated *with* 143 *Diagrams.* 6/- net; 6/4 post free.

**This handbook has been specially designed to meet the requirements of Nurses entering for the C.M.B. Examinations, and its use as a text-book should do much to ensure success.**

**HOW TO BECOME A CERTIFIED MIDWIFE.**
By E. L. C. Appel, M.B., B.S., B.Sc. Lond. Crown 8vo., bound limp cloth, 1/- net; 1/1 post free.

**A PRACTICAL HANDBOOK OF MIDWIFERY.**
By Francis W. Haultain, M.D.
New and Revised Edition. Profusely illustrated with Original Cuts, Tables, &c.
Handsomely bound in leather, 6/- net; 6/3 post free.

**SIMPLE INTRODUCTORY LESSONS ON MIDWIFERY.**
By M. Loane. Crown 8vo., illustrated cloth, 1/- net; 1/1 post free.
A most useful work to Nurses beginning the theoretical study of Midwifery. It also explains the meaning of most of the technical words connected with the subject.

**NURSING NOTES ON MIDWIFERY AND GYNÆCOLOGY.**
By Sister Ross, Rotunda Hospital, Dublin. 2/- net; 2/1 post free.
(Suitable size for apron pocket.)

**THE MIDWIVES' POCKET BOOK** (with Prefatory Chapter on the Midwives Bill).
By Honnor Morten. Small crown 8vo. cloth, 1/- post free.

**INSTRUCTIONS TO MIDWIVES.**
Compiled by the Midwives' Supervising Committee of the Corporation of Manchester.
Crown 8vo., 6d. net; 7d. post free.

**LECTURES ON MIDWIFERY.**
By E. Stanley Hoare, M.R.C.S., D.P.H. Lond.
Based upon the Syllabus of the Central Midwives Board. 3/6 net; 3/9 post free.
Complete in cloth portfolio, 4/6 net; 4/9 post free.

***Catalogue of Nursing Manuals sent post free on application.***

**The Scientific Press, Ltd.,**

**Publishers of Nursing Text-Books, Charts, and Case-Books of all Descriptions.**

**28 & 29 SOUTHAMPTON STREET, STRAND, LONDON, W.C.**

